# Recurrence of Left Ventricular Outflow Tract Obstruction Requiring Alcohol Septal Ablation after Transcatheter Aortic Valve Implantation

**DOI:** 10.1155/2018/5026190

**Published:** 2018-12-02

**Authors:** Hideki Kitahara, Kaoru Matsuura, Atsushi Sugiura, Akiko Yoshimura, Takahiro Muramatsu, Yusaku Tamura, Takashi Nakayama, Yoshihide Fujimoto, Goro Matsumiya, Yoshio Kobayashi

**Affiliations:** ^1^Department of Cardiovascular Medicine, Chiba University Graduate School of Medicine, Chiba, Japan; ^2^Department of Cardiovascular Surgery, Chiba University Graduate School of Medicine, Chiba, Japan; ^3^Department of Anesthesiology, Chiba University Graduate School of Medicine, Chiba, Japan

## Abstract

Left ventricular outflow tract (LVOT) obstruction is sometimes observed in patients with severe aortic stenosis (AS). It is still controversial how to manage the remaining severe AS, when LVOT obstruction is well-controlled by medical therapy. We report a case with acute recurrence of LVOT obstruction requiring emergent alcohol septal ablation (ASA) after transcatheter aortic valve implantation (TAVI), even in a stable state on beta-blockers. For the ASA procedure, transesophageal echocardiography was useful to clearly observe the perfusion area of the target septal branch by injecting microbubble contrast. Since it took some time to cause the recurrence of LVOT obstruction in this case, careful evaluation should be done after TAVI in high-risk patients for LVOT obstruction before terminating the TAVI procedure.

## 1. Introduction

Left ventricular outflow tract (LVOT) obstruction is sometimes observed in patients with severe aortic stenosis (AS) [1]. If a patient has both conditions, surgical aortic valve replacement (SAVR) with septal myectomy or, alternatively, alcohol septal ablation (ASA) followed by transcatheter aortic valve implantation (TAVI) should be considered [2, 3]. On the other hand, in cases with improved LVOT obstruction by medical therapy, it is still controversial how to manage the remaining severe AS in high surgical risk or inoperable patients. We report a case with acute recurrence of LVOT obstruction requiring ASA after TAVI.

## 2. Case Presentation

**Figure 1:**
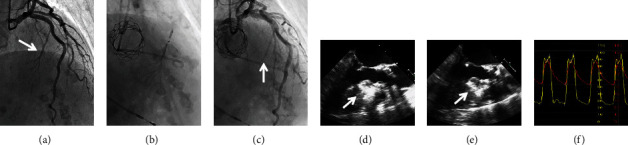
(a) The first septal branch was confirmed beforehand. (b) Tip injection into the septal branch using a 2 mm balloon. (c) Total occlusion of the septal branch was confirmed after alcohol injection. (d) Transesophageal echocardiography confirmed the perfusion area of the septal branch as a bright area by contrast injection. (e) Alcohol was administered into the septal branch. (f) Finally, pressure gradient at the left ventricular outflow tract was decreased.

A frail 86-year-old female, presenting with dyspnea on exertion with elevated brain-type natriuretic peptide (BNP) level of >900, at high surgical risk (the Society of Thoracic Surgeons risk score 9.8%), was referred to our institution to consider treatment for severe AS. Transthoracic echocardiography (TTE) revealed not only severe AS (aortic valve area was 0.58 cm^2^, and peak velocity was 4.0 m/s) but also diffuse left ventricular hypertrophy except posterior wall (Maron type III hypertrophic cardiomyopathy) and LVOT obstruction with systolic anterior motion (SAM) of the mitral valve, leading to moderate mitral regurgitation (MR). Peak velocity was 2.9 m/s, and mean pressure gradient was 32 mmHg at the LVOT during the Valsalva maneuver. After administration of a beta-blocker (bisoprolol 1.25 mg/day), LVOT obstruction and SAM disappeared, and MR was reduced to mild degree. Since peak velocity across the aortic valve and BNP level were still high, TAVI was planned to treat AS. Coronary angiography confirmed the first major septal branch perfusing the basal septum (Figure 1(a)). Thus, ASA was considered as a rescue option for recurrence of LVOT obstruction.

**Figure 2:**
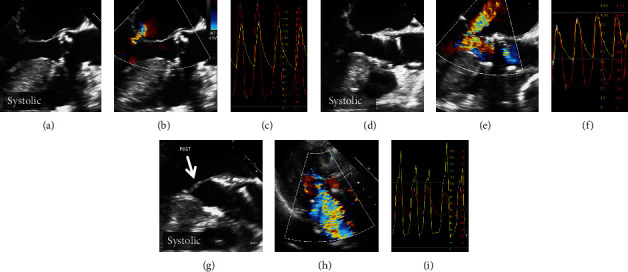
(a–c) No systolic anterior motion (SAM) was observed before transcatheter aortic valve implantation (TAVI). (d–f) Five minutes after TAVI, there was no SAM or pressure gradient. (g) Fifteen minutes later, SAM clearly emerged. (h) Transthoracic echocardiography showed severe mitral regurgitation. (i) Pressure gradient was >50 mmHg at the left ventricular outflow tract.

The 23 mm SAPIEN 3 valve (Edwards Lifesciences, Irvine, CA, USA) was successfully implanted via transfemoral approach with general anesthesia and transesophageal echocardiography (TEE) guidance, under continuous infusion of beta-blocker (landiolol) and volume load. Five minutes after implantation, there was no obvious LVOT obstruction and SAM (Figures 2(d)–2(f)). However, 15 minutes later, TEE clearly showed SAM and severe MR, and pressure gradient was >50 mmHg at the LVOT, even under increased dose of beta-blockers (Figures 2(g)–2(i)). Because the patient's hemodynamic status became rapidly unstable, we decided to perform emergent ASA. Through a 2 mm over-the-wire balloon, microbubble contrast was injected into the first septal branch to confirm its perfusion area in the septum, which was located on the opposite side of SAM, using TEE (Figure 1(d)). After injecting a total of 3.5 ml absolute alcohol into the septal branch (Figure 1(e)), total occlusion was confirmed by coronary angiography (Figure 1(c)), and the pressure gradient at the LVOT was decreased below 10 mmHg (Figure 1(f)). Final TEE and TTE showed no LVOT obstruction or SAM and reduced MR. After ASA, complete atrioventricular block occurred, and a permanent pacemaker was implanted 3 days later. At 6-month follow-up, TTE showed good bioprosthetic valve function, regional hypokinesis of the basal-mid septum, and no evidence of LVOT obstruction.

## 3. Discussion

In patients with severe AS accompanied by LVOT obstruction, SAVR with concomitant septal myectomy should be considered [2]. For high surgical risk or inoperable patients, preceding ASA followed by TAVI may be an alternative strategy [3]. However, LVOT obstruction can often be well-controlled by medical therapy. In our case, because the LVOT obstruction was eliminated with a low-dose beta-blocker, TAVI was performed for the remaining AS. As a result, LVOT obstruction recurred despite sufficient beta-blocker infusion and volume load, as preoperatively concerned. Even in a stable state on beta-blockers, LVOT obstruction can occur, possibly by rapid reduction of left ventricular afterload and accelerated blood flow after TAVI.

ASA is a good option to recover from acute deterioration of LVOT obstruction after TAVI, because most patients are at high surgical risk or inoperable [4, 5]. For the ASA procedure, TEE may be useful to clearly observe and confirm the perfusion area of the target septal branch by injecting microbubble contrast, if the patient undergoes TAVI with general anesthesia and TEE guidance. In addition, it is noteworthy that it took some time to cause the recurrence of LVOT obstruction and SAM in this case, although neither were detected immediately after valve implantation. Transient left ventricular dysfunction due to rapid pacing might be one of the reasons for the delayed appearance of LVOT obstruction and SAM after TAVI.

## 4. Conclusions

LVOT obstruction can recur after TAVI even in a stable state on beta-blockers. ASA with TEE guidance is a good option to recover from acute deterioration of LVOT obstruction after TAVI. Careful observation should be done in high-risk patients for LVOT obstruction before terminating the TAVI procedure.

## References

[1] Bach D. (2005). Subvalvular left ventricular outflow obstruction for patients undergoing aortic valve replacement for aortic stenosis: echocardiographic recognition and identification of patients at risk. *Journal of the American Society of Echocardiography*.

[2] Kayalar N., Schaff H. V., Daly R. C., Dearani J. A., Park S. J. (2010). Concomitant septal myectomy at the time of aortic valve replacement for severe aortic stenosis. *The Annals of Thoracic Surgery*.

[3] Sayah N., Urena M., Brochet E., Himbert D. (2018). Alcohol septal ablation preceding transcatheter valve implantation to prevent left ventricular outflow tract obstruction. *EuroIntervention*.

[4] Krishnaswamy A., Tuzcu E. M., Svensson L. G., Kapadia S. R. (2013). Combined transcatheter aortic valve replacement and emergent alcohol septal ablation. *Circulation*.

[5] Yanagiuchi T., Tada N., Mizutani Y., Matsumoto T., Sakurai M., Ootomo T. (2017). Feasibility assessment of alcohol septal ablation in transcatheter aortic valve replacement using multidetector computed tomography. *JACC: Cardiovascular Interventions*.

